# Sensing the storm: how inflammatory signalling drives reactivation of the human cytomegalovirus major immediate-early promoter

**DOI:** 10.1099/jgv.0.002276

**Published:** 2026-06-30

**Authors:** Benjamin A. Krishna

**Affiliations:** 1 Department of Medicine, University of Cambridge, Cambridge, CB20QQ, UK

**Keywords:** HCMV, latency, MIEP, promoter, reactivation

## Abstract

Seventy years after the discovery of human cytomegalovirus (HCMV), perhaps its most influential contribution to molecular biology remains the major immediate-early promoter (MIEP). A modified form of this powerful regulatory element is now used ubiquitously in biotechnology to drive protein expression, yet within the virus itself, the full promoter performs a far more complex task: governing the balance between latency and lytic replication. This review discusses our understanding of the transcription factors that regulate MIEP activation and repression and how this regulatory network shapes HCMV latency and reactivation.

## The MIEP as a complex switch between latency and reactivation

Human cytomegalovirus (HCMV), a β-herpesvirus, infects the majority of the global population and is typically asymptomatic in immunocompetent hosts [1]. Its clinical importance is in immunocompromised, immunosuppressed and immunonaïve individuals, where viral reactivation or primary infection can cause significant morbidity [2]. Lifelong persistence is underpinned by latency, where viral gene expression is highly restricted and no virus is produced; limited viral gene expression also restricts antigen presentation, thereby helping to evade host immunity [3]. The restricted minority of genes which are expressed, however, skew immune activation towards regulatory T cells [4,5]. This phenomenon may be due to the production of viral interleukin (IL)-10 during latency [6], as well as the induction of cellular IL-10 and transforming growth factor-β in latently infected cells [7]. These cytokines induce a microenvironment around latently infected cells which also suppresses T cell activation [7]; which has recently been observed in ex vivo bone marrow-derived T cells [8]. This process of latency or lytic infection is cell-type dependent: CD34^+^ stem cells and CD14^+^ monocytes are sites of latency while other cell types, including monocyte-derived macrophages [9] and dendritic cells [10], support viral replication.

Latency occurs due to a tightly regulated switch between latent and lytic viral transcriptional programmes. Central to this switch is the major immediate-early promoter (MIEP), which controls expression of UL122 (IE2) and UL123 (IE1). Because IE1 and IE2 transactivate early viral promoters, regulation of the MIEP determines progression into the lytic transcriptional cascade. Interestingly, IE2 itself can bind the MIEP in a cis-repression site at late times of infection [11,15], presumably to prevent unnecessary transcription from the MIEP once all viral genes are activated.

**Fig. 1.**
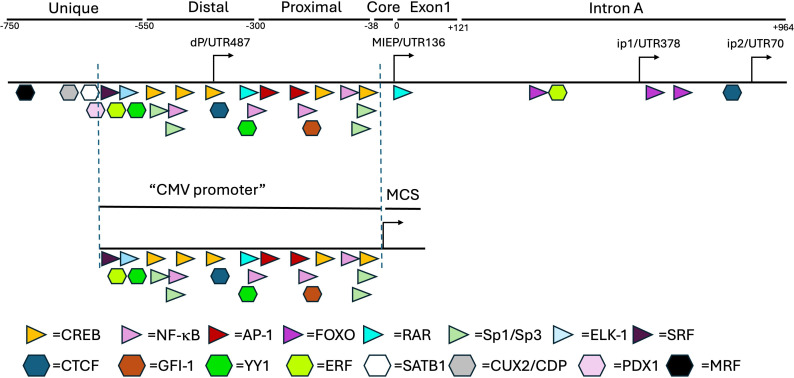
Schematic of the HCMV MIEP and the truncated ‘CMV promoter’ used in expression vectors. Top: The full HCMV MIEP (TB40e) is organized into distinct functional regions: Unique, Distal enhancer, Proximal enhancer, Core, Exon 1 and Intron A. Four known transcription start sites are indicated (dP/UTR487, MIEP/UTR136, iP1/UTR378 and iP2/UTR70 [120,121]). Numbers indicate positions relative to the canonical transcription start site. Binding sites for published transcription factors are depicted as coloured shapes and mapped to their approximate positions within the locus. Bottom: The ‘CMV promoter’ commonly used in expression plasmids comprises only the proximal and distal enhancer regions upstream of multiple cloning site, and the presence of other transcription start sites is not known. The following binding motifs were identified: CREB (cyclic AMP response element-binding protein, TGACGTCA); NF-κB (nuclear factor kappa B, GGAAAKYCCC); AP-1 (activator protein−1, TGA(C/G)TCA); FOXO (Forkhead box O, NNAAA(T/C)AA); RAR (retinoic acid receptor, TGACC); Sp1/Sp3 (specificity factor, 1/3, GGGCGG); ELK1 (erythroblast transformation specific like protein, GAGTTCCGCG); SRF (serum response factor, GCCCATATATGG); CTCF (CCCTC-binding factor, GCCC(T/C)C(T/C)A); GFI (growth factor independence 1, GAAATCCCCGTG); YY1 (Yin Yang 1, GGCCATTT); ERF (ETS2 repressor factor, CCG(G/T)AA); SATB1 (special AT-rich sequence-binding protein 1, TATTAATAA); CUX2/CDP (CCAAT, displacement protein, ATTGGNNNNNNNCCAAT); PDX1 (pancreatic and duodenal homeobox 1, CTAAT); MRF (myogenic regulatory factor, ATATCGATAT).

The HCMV MIEP, first mapped and functionally characterized in the 1980s [16,19], spans roughly one kilobase upstream of the IE transcriptional start site and comprises enhancer, unique and modulator regions that are rich in transcription factor binding motifs [18]. The MIEP is by some margin the largest promoter in the HCMV genome and more complex. Unlike the truncated ‘CMV promoter’ widely used in expression systems (Fig. 1), the full viral MIEP coordinates both activating and repressive transcription factor binding sites. But why? Over three decades of research have revealed a promoter evolutionarily tuned to respond to inflammatory signalling, cellular environment and differentiation state of the cell.

The evolutionary pressure for the MIEP to respond to inflammatory signalling is evident by the transcription factor motifs contained within. Upon viral entry, host innate immune pathways are rapidly activated [20], particularly nuclear factor kappa B (NF-κB) [21,22]. Because the MIEP must be activated to initiate the lytic transcriptional cascade, co-opting these early antiviral signals provides an efficient mechanism for triggering immediate-early gene expression. In this way, HCMV exploits host inflammatory responses to promote its own replication.

The same principle applies during reactivation. Inflammatory cues are enriched in tissues where monocytes differentiate into macrophages or dendritic cells. By encoding responsiveness to these signals within the MIEP, HCMV has evolved to ensure that reactivation occurs preferentially in peripheral tissues rather than in circulation, which is advantageous for viral dissemination onto new hosts. The presence of inflammation in models of organ transplantation [23] could help explain why HCMV reactivation is both so quick and so common in the immunosuppressed transplant patient.

Importantly, the MIEP exhibits responsive heterogeneity: it can respond to multiple signalling pathways, allowing reactivation to proceed through different inflammatory or differentiation-associated cues. This responsive heterogeneity likely maximizes the probability that an appropriate environmental signal will trigger reactivation, enhancing the virus’ capacity for transmission to a new host. Interestingly, different transcription factors play roles in different contexts and can compensate for each other [24]. This process appears to require multiple concomitant signals as well, for example, in reactivation from IL-6 treated, monocyte-derived dendritic cells, extracellular signal-regulated kinase, mitogen stimulated kinase and cyclic AMP response element-binding protein (ERK-MSK-CREB) mediates MIEP de-repression, but a Src family kinase signal to recruit MOZ, a histone acetyltransferase (HAT), is also needed to drive opening of chromatin [25].

Consistent with this responsive heterogeneity, deletion of individual transcription factor binding elements within the MIEP, including all four NF-κB sites [26], both AP-1 sites [27] and the ATF/CREB elements [28] can have minimal impact on lytic replication in many cell types, with compensatory contributions from neighbouring elements, such as Elk-1/serum response factor (SRF) [24]. This dense placement of overlapping inputs likely reflects an evolutionary pressure to activate the MIEP transcriptionally across diverse cellular and physiological contexts.

Thus, rather than functioning like the constitutively active CMV promoter found in plasmids, the MIEP operates as a complex, signal-responsive regulatory element whose activity determines whether the virus remains silent or enters productive replication [29]. This is in part because the plasmid CMV promoter contains only the proximal and distal enhancer regions, removing the unique region which contains many repressive elements, as well as a repressive CCCTC-binding factor (CTCF) site in intron A (Fig. 1). Although not the focus of this review, the HCMV genome contains many viral genes which aid in this process, repressing the MIEP during latency or amplifying activating signals to facilitate MIEP de-repression [3].

## Cell-type dependence of MIEP activity

MIEP-driven transcription is strongly cell-type dependent. In permissive, differentiated cells, including fibroblasts, endothelial cells, epithelial cells and fully differentiated myeloid cells, HCMV infection results in rapid and robust immediate-early gene expression, initiating the lytic transcriptional cascade and productive replication [30,31]. However, even among permissive cell types, levels of expression and responsiveness to specific stimuli vary considerably [26,32,39].

In contrast, in undifferentiated myeloid cells: bone marrow resident CD34^+^ haematopoietic progenitor cells (HPCs) and circulating CD14^+^ monocytes, viral gene expression and particularly IE gene expression is repressed. This was demonstrated both in early transient transfection assays [40,43] and in infected primary cells [10,44, 45]. That this phenotype is seen in both models is important as the plasmid CMV promoter lacks many repressive transcription factor binding sites (Fig. 1). This shows that a fundamental lack of positive transcription factors in myeloid progenitor cells is a key factor in diminished MIEP activity and subsequent HCMV latency. Reduced MIEP activity in these cells reflects differences in transcription factor availability and occupancy, as well as changes in chromatin structure. This model, therefore, places the MIEP as a molecular switch whose activity is dictated by host-cell identity, external signalling factors and differentiation state.

## Chromatin structure as the foundation of MIEP activity

Upon viral entry into cells, the viral genome is rapidly chromatinized in both lytic and latent infection [46,47] by Daxx, with an unusual histone H3 composition [48] that varies by cell type [39]. In undifferentiated myeloid cells, chromatin immunoprecipitation studies have demonstrated enrichment of repressive histone marks, including H3K9me3 and H3K27me3, together with heterochromatin protein 1 (HP1) and hypoacetylated histone H4 at the MIEP [49,52]. In this configuration, RNA polymerase II access to the core promoter is restricted, establishing a ‘default-to-silence’ state that prevents initiation of the lytic transcriptional programme.

Control of MIEP activity ultimately reflects the balance between activating and repressive histone marks deposited by HATs, histone deacetylases (HDACs), histone methyltransferases (HMTs) and chromatin remodellers, such as FACT (facilitates chromatin transcription) [53]. These enzymes are recruited and regulated by transcription factors [32,33, 54], linking signal-dependent transcription factor activity directly to chromatin state. In a resting monocyte or CD34^+^ cells, inducible activators, including NF-κB and CREB remain inactive or are insufficiently recruited to overcome this epigenetic barrier. Thus, latency reflects both active enforcement of a repressive chromatin architecture as well as the absence of activation signals.

HCMV itself modulates the balance of chromatin remodellers to maintain latency: infected monocytes secrete cytokines [7], which upregulate HDAC expression [55]. HCMV also expresses lncRNA4.9 (long non-coding RNA 4.9) during latency which may enhance H3K27me3 markers [56].

## Signal-driven chromatin remodelling during reactivation

Due to the maintained repressive state during latency, MIEP de-repression requires coordinated reversal of chromatin structure via transcription factor binding. This allows the MIEP to respond to external cues, such as inflammatory signals as well as transcription factor availability, which is cell type dependent.

In this framework, transcription factors function primarily as modifiers of chromatin architecture rather than simple binary switches. Repressors stabilize heterochromatin, while activators recruit enzymes that open chromatin and permit transcription. The balance of these opposing forces, determined by cell type and signalling environment, dictates MIEP activity and viral behaviour.

## Repressive transcription factors and silencing of the MIEP

### CCCTC-binding factor

A growing body of evidence identifies CTCF as a critical regulator of the MIEP. Initial studies demonstrated that CTCF binds a conserved motif within intron A of the MIE locus (Fig. 1) during lytic infection, where it negatively regulates MIEP transcription and viral replication, likely by modulating RNA polymerase II activity rather than alternative splicing [57]. More recent work extended these observations to latency, showing that CTCF is upregulated in latently infected monocytes and enriched at the MIEP in a US28-dependent manner, contributing to suppression of MIEP transcription [58]. Chromosome conformation capture approaches demonstrated that CTCF binding is not limited to a single site: two CTCF-binding motifs, one within intron A and one in the enhancer (Fig. 1), create a chromatin loop across the MIE locus during latent infection [59]. This loop represses transcription while disruption of the enhancer CTCF site abrogates loop formation and impairs the establishment or maintenance of latency in myeloid cells [59]. Upon cellular differentiation and viral reactivation, CTCF protein levels and occupancy decline, the loop is resolved and MIE transcription is derepressed [59]. These findings are the first to show that a three-dimensional regulatory mechanism helps balance latency and reactivation and highlights that HCMV, like other herpesviruses, exploits host genome architectural machinery to fine-tune promoter activity [59,60]. How this repressive chromatin looping is released during reactivation remains unclear. As US28 appears to play a role in CTCF activation, perhaps the ability for US28 to change signalling in a cell type dependent manner [61] also triggers release of the MIEP from CTCF-mediated chromatin looping.

### Yin Yang 1

The zinc-finger transcription factor Yin Yang 1 (YY1) was among the first cellular transcriptional repressors shown to bind the MIEP. Early work by Liu *et al*. [62] identified YY1 binding to motifs within the distal enhancer as a repressive factor (Fig. 1), and also showed that YY1 expression decreases after T2 cell differentiation [62], however, the same was not seen during monocyte differentiation [10]. YY1 association with the MIEP is activated by signalling via BMPR2 (bone morphogenetic protein receptor 2) [63]. As the name suggests, YY1 can both activate and repress gene expression in a context-dependent manner, so how YY1 represses MIEP expression in the context of HCMV latency in monocytes remains unclear. Additionally, YY1 activates pro-inflammatory gene expression in a cooperative manner with both CREB and NF-κB [64,66], which are both activated under many reactivation conditions [67], meaning YY1 might facilitate reactivation as well. Knock out of YY1 in Induced Pluripotent Stem Cells does not appear to have a large effect on reactivation potential [63], but only an inducible YY1 system would be able to answer this question fully by allowing knock out of YY1 concomitant with reactivation signals. As YY1 also contributes to chromatin looping [68], how this might interplay with CTCF-mediated looping would be an interesting future area of research.

### ETS2 repressor factor

ETS2 repressor factor (ERF) recognizes Ets consensus sequences (Fig. 1). In undifferentiated, non-permissive cells, such as NTera-2, ERF recruits HDAC1 to the MIEP, inducing chromatin condensation and transcriptional repression; this repression is not observed in differentiated cells with lower HDAC1 expression. Together, these findings suggest that interactions among ERF and HDAC1 also help shape MIE locus architecture [69,70]. Whether this mechanism is seen in primary cell models of HCMV latency is unclear.

### KAP1/TRIM28 and associated chromatin repressors

KRAB-associated protein 1/Tripartite Motif-containing protein 28 (KAP1/TRIM28) acts as an epigenetic repressor on the latent HCMV genome by recruiting HP1 and the HMT SETDB1 to induce H3K9 trimethylation and transcriptional silencing of viral immediate-early genes in CD34^+^ hematopoietic stem cells [71]. Interestingly, KAP1 does not bind near the MIEP but presumably acts at a distance [71], which occurs via spreading of H3K9me3 and HP1 β across long ranges of DNA [72,73]. Upon differentiation or during productive infection, mTOR (mechanistic target of rapamycin)-mediated phosphorylation of KAP1 inhibits its repressive activity, allowing chromatin opening and enabling viral gene expression and reactivation [71].

### Promyelocytic leukaemia nuclear body suppression of the MIEP

In all cells, the incoming HCMV genome is rapidly targeted to promyelocytic leukaemia (PML) nuclear bodies (also known as nuclear domain or ND10 bodies), where core components like Sp100 and Daxx act as an intrinsic restriction system that generates repressive chromatin and silences transcription from the MIEP [74]. In lytic settings, the tegument protein pp71 is brought to PML via Daxx, allowing MIEP activation via degradation of hDaxx and displacement of ATRX (alpha-thalassemia/mental retardation X-linked) [75,77]. During latency, however, it is suggested that pp71 is not trafficked, allowing PML bodies to persist and causing MIEP repression [78]. Consistent with this, in THP-1 cells, siRNA knockdown of hDaxx can relieve IE gene repression in laboratory-adapted HCMV. However, siRNA knockdown of Daxx in THP-1 or T2 cells did not show an increase in MIEP transcription when clinical HCMV strains were used [79,81]. This could be due to the expression of other factors from the ULb’ region which provide additional repression of the MIEP [82,83]. During lytic infection, IE1 may inhibit SUMOylation of PML [84], while Latency Unique Nuclear Antigen (LUNA) encodes a deSUMOylase which helps disperse PML bodies to facilitate HCMV reactivation [85]. That LUNA is expressed during latency suggests it is expressed pre-emptively to remove PML bodies which would otherwise be inhibitory to MIEP upon reactivation [85]. Additionally, as disruption of LUNA does not adversely affect latent viral carriage, this work suggests that PML bodies are not playing a role in long-term HCMV genome repression.

## Activating transcription factors and reactivation of the MIEP

### NF-κB

The four binding sites for the transcription factor NF-κB were some of the first found in the MIEP [21,36, 86]. Activation of NF-κB through the canonical IκB kinase pathway leads to nuclear translocation of various homo/heterodimers that can occupy these sites and stimulate transcription. This NF-κB response is a classic inflammatory response which occurs both immediately after viral entry [21,87] and also in inflamed tissues [67], facilitating both viral lytic replication and reactivation from latency. Indeed, in monocyte-derived macrophages, non-canonical activation of NF-κB appears to occur via p52/Bcl-3, not p50/p65 as seen in fibroblasts [88]. This suggests that HCMV can exploit NF-κB to reactivate in myeloid cells without activating innate immune functions which would be detrimental to viral replication.

Interestingly, deletion of NF-κB binding sites from the MIEP does not affect reactivation from latency when dendritic cells are treated with IL-6 [89]. NF-κB does, however, reactivate HCMV under redox stress [90], and in murine transplant and inflammation models [91,95]. This is a recent example of how the MIEP has likely evolved to allow HCMV reactivation under different contexts.

NF-κB activation is commonly associated with TNF-α signalling. TNF-α stimulates HCMV activity in monocytic cell lines and reactivation correlated with clinical situations where TNF-α is present [96], while treatment with TNF-α activates the MIEP via NF-κB [97,98]. The TNF-α receptor 1 (TNFR1) is upregulated by UL138 in fibroblasts [99] and during latency [100], which hypersensitizes infected cells to facilitate reactivation. TNFR2 is upregulated in fibroblasts via UL148 and UL148D [101]. TNFR signalling activates the MIEP via NF-κB [102].

Both US28 and LaCMV-IL-10 (a viral IL-10 homologue expressed during latency [6]), and miRNAs UL122-3p and US5-1 repress NF-κB activation during latent infection [6,61, 103, 104]. The HCMV lncRNA2.7 also blocks the production of reactive oxygen species, which also induce NF-κB [90].

### CREB and ATF family members

There are five CREB binding sites within the MIEP (Fig. 1) [105]. CREB binds the MIEP, even during latency, but requires activation by phosphorylation at Ser133 through mitogen- and stress-activated kinase (MSK1/2), downstream of ERK and p38, but requiring only ERK [89]. Early transient transfection work showed these elements to be responsive and drive MIEP activity [106,108]. Unlike NF-κB, these elements do not play a role in activating the MIEP in fibroblasts or NTera2 (NT2)-derived neuronal cells [28]. However, CREB activation by forskolin [109] or vasoactive intestinal peptide [110] can activate the MIEP in NT2 precursor cells. In latently infected primary monocytes differentiated with IL-4 and GM-CSF followed by IL-6 or lipopolysaccharide, ERK1/2-MSK-CREB plays a central and necessary role in MIEP de-repression [89]. The specific role of CREB binding the MIEP in this model is for the recruitment of the kinase activity of MSKs to initiate the chromatin remodelling at the MIEP required for reactivation. During reactivation, UL33 also appears to activate CREB which drives MIEP activation [111,112].

### Activator protein-1

The activator protein-1 (AP-1) complex, typically composed of c-Fos and c-Jun dimers, is a classic factor associated with inflammation. AP-1 is canonically activated by PKC-MEK-ERK (protein kinase C-mitogen-activated protein kinase kinase-extracellular signal-regulated kinase, but was initially discovered as a factor activated by phorbol 12-myristate 13-acetate (PMA/TPA) [113,114]. AP-1 was identified as a crucial activator of the MIE enhancer [115]; two functional AP-1 motifs within the enhancer respond to phorbol ester stimulation [27]. In a model of murine CMV (MCMV) infection, these sites could be deleted with no effect on MCMV virulence but were important when NF-κB sites were also deleted [27]. In contrast to the MCMV model, the AP-1 binding sites are necessary for MIEP de-repression from latency in Kasumi-3 and CD34 +HPC models when reactivated by PMA stimulation [116]. In agreement with this, AP-1 also binds the MIEP in murine models of transplantation [91]. HCMV US28 represses AP-1 activation to maintain latency in THP-1 cells [117] via repression of the mitogen-activated protein kinase-Src signalling pathway in cord blood-derived monocytes [118]. Indeed, as Src activation by IL-6 treatment of dendritic cells [25] is necessary for HCMV reactivation in this model, it is tempting to speculate that US28-mediated repression of Src family kinase signalling during latency and IL-6-mediated activation of the Src family member HCK (hematopoietic cell kinase) during reactivation represent two sides of the same coin. In this model, US28 attenuates HCK activity to prevent premature reactivation in undifferentiated myeloid cells [119], while differentiation to a dendritic cell phenotype coupled with inflammatory IL-6 signalling overcomes this block.

### The intronic transcription start sites

Within the first intron (intron A) of the MIE coding region are two alternative transcription start sites which produce transcripts commonly termed iP1 and iP2, or UTR378 and UTR70, respectively [120]. These transcripts were initially discovered in MRC-5 fibroblasts during lytic infection and, along with a number of transcripts upstream of the MIEP canonical start site, all encode full-length UL122 and UL123 transcripts [120].

The iP2 transcript is expressed at higher levels than transcripts from the canonical MIEP during latency in THP-1 cells [121] and Kasumi-3 cells [116] and show strong responsiveness to PMA induced HCMV reactivation in both systems [116,121]. Although the fold changes in iP1 are large [121], when absolute transcript levels are calculated using a standard curve, the amounts of iP1 transcript are low compared to MIEP and iP2 transcripts [116].

Deletion of the intronic region encoding the iP1 and iP2 start sites also abrogated HCMV reactivation from latency in primary CD34 +HPCs [121]. However, the iP2 transcript did not show this responsiveness in dendritic cells derived from either CD34 +HPCs or CD14 +monocytes when treated with IL-6, nor in CD14 +monocytes treated with HDAC inhibitors (HDACi), but did show responsiveness when CD14 +monocytes were treated with PMA [122].

Initial work in this area hinted that there might be a reciprocal relationship between transcription of the canonical MIEP and iP2. Deletion of the MIEP core promoter from a plasmid containing the MIE genomic locus did not completely abrogate IE1 and IE2 expression, instead resulting in increased expression of the alternative MIE transcripts [120]. IE2 is also known to repress MIEP activity at late times of infection [14], the same time that iP2 transcripts increase in abundance [120]. Infection with a recombinant virus unable to auto-repress the MIEP resulted in impaired iP2 transcription [123].

This reciprocal activity was also reflected during reactivation in monocyte-derived dendritic cells: in cells where the MIEP remained heavily silenced, iP2 activity was detectable at very early times post-reactivation, whereas robust activation of the MIEP was associated with reduced or delayed iP2 transcription [123]. Importantly, inhibition of IL-6-dependent signalling pathways known to drive MIEP de-repression in dendritic cells preferentially inhibited the canonical MIEP and not the iP2 promoter [123].

Together, these data suggest that the relative transcription from the canonical MIEP and iP2 during both lytic infection and reactivation is dictated by the extent of MIEP silencing in a given cell type and the nature of the reactivation stimulus encountered. Alternative transcription start sites may exist as a failsafe for HCMV reactivation from latency, allowing reactivation even if the MIEP has been repressed too strongly by the factors discussed above. Alternatively, as different stimuli appear to activate transcription from different sites, iP2 and others may have been selected to broaden MIEP responsiveness and provide ‘multiple escape routes’.

### FOXO transcription factors and other factors binding within intron A

The Forkhead box O (FOXO) family members are currently the only transcription factors with binding sites unique to the intron A region and appear to activate transcription from the intronic start sites preferentially [124]. Deletion of these sites reduces HCMV reactivation potential from CD34^+^ HPCs [124]. Interestingly, FOXO factors also promote apoptosis and so must be carefully regulated via UL7 [125].

Although other transcription factors, such as AP-1 activate transcription from these intronic start sites [116], they do not have binding sites within intron A itself. There is a functional CTCF binding site within intron A [59] but its role in controlling transcription from the intron itself remains unclear. Whether other transcription factors are unique to intron A, why some appear to be localized while others are not, and how they might co-operate with factors like CTCF, are unclear.

### The MIEP as a target for HCMV therapies

Understanding which transcription factors regulate the MIEP has major implications for controlling HCMV latency and reactivation in clinical settings. This approach could take two forms: ‘kick and kill’, where MIEP repression is inhibited, triggering viral gene expression in otherwise latently infected cells [126] and their subsequent detection and killing by the high numbers of HCMV-specific cytotoxic T lymphocytes routinely present in normal healthy HCMV carriers [127]. This approach could be used in healthy individuals before immune suppression or organ donation to reduce the viral load and risk of subsequent HCMV disease.

Such ‘kick and kill’ strategies have included the use of HDACi on latently infected cells, which induce viral MIEP activation sufficiently to induce killing of latently infected cells by host T cells *in vitro* [55,128,130]. *In vivo*, patients taking the HDACi valproic acid have lower HCMV latent loads [131]. Whether inhibition of DNA methyltransferases [132,133] might work in a similar fashion is unclear. Interestingly, MIEP activity after HDACi treatment is dependent on CREB and NF-κB elements in the MIEP [134].

Conceptually similar, bromodomain proteins, a class of protein which detects histone acetylation and facilitates gene expression, have been highlighted as having a role in HCMV reactivation. Inhibition of these proteins appears to activate the MIEP and expression of IE1, but without the expression of virally encoded immune evasion molecules expressed in the lytic cycle or the production of infectious virions, thereby revealing the otherwise latently infected cells to the immune system without the risk of immune evasion or full reactivation [131].

Alternatively, inhibition of activator signals could prevent MIEP de-repression, keeping HCMV in a latent state. Examples of this include NF-κB [37] AP-1 [116] or CREB [89] inhibitors. These ‘block and lock’ approaches are less well studied as viable clinical therapeutics, but can block HCMV reactivation in ex vivo cell culture models [37,89, 116]. Naturally, as the inhibition of transcription from the MIEP leads to transcription from alternate start sites [123], a therapeutic which blocks transcription broadly from the MIEP region would need to be developed first.

## Conclusions

The transcription factor binding landscape of the MIEP is not arbitrary. Rather, it likely reflects the dual evolutionary pressures that have shaped the HCMV genome over millennia of co-evolution with its human host. For the virus to initiate productive replication, the MIEP must be activated at immediate-early timepoints in a newly infected cell – an environment characterized by rapid innate immune activation and the induction of pro-inflammatory signalling. For the virus to reactivate from latency, the same promoter must respond but without virus binding and entry to trigger inflammatory signals. As a result, HCMV reactivation occurs in inflamed tissue after myeloid differentiation, which provides signals to the MIEP in the absence of virus binding. During latency, however, there is evolutionary pressure not to express immunodominant MIE antigens, and so factors associated with myeloid differentiation status are also selected. The consequence of this selective pressure is a promoter architecture dominated by binding sites for transcription factors associated with inflammation: NF-κB, AP-1 and myeloid lineage commitment: retinoic acid receptors (RARs), CREB, FOXO, growth factor independence (GFI), MOZ and YY1.

In some ways, it is perhaps surprisingly wasteful for HCMV to require an inflammatory signal to reactivate efficiently. Perhaps, searching the MIEP for additional transcription factor binding motifs may still reveal new mechanisms of HCMV reactivation known to the virus but not us. Recently CTCF has been the focus of most work on MIEP repressors in latency [58,60], after a long period with only sparse publications [57]. This raises the exciting question of which transcription factor might emerge from the fringes to become the new hot topic. Some under-studied factors include reporter assay data for CUX1/CDP [135], binding sites for SAT1B, SBP/HMGB [136], GFI-1 [137] and myogenic regulatory factor (MRF) [138].

In this light, the MIEP is less a conventional promoter than an exquisitely tuned sensor of host physiological state, evolved to couple viral gene expression to precisely those cellular contexts in which replication or dissemination is most likely to succeed. Understanding the MIEP through this evolutionary lens not only clarifies why so many transcription factors converge on this single regulatory element, but also highlights why therapeutically targeting this convergence point remains one of the most promising and underexplored strategies for controlling HCMV disease.

## Abbreviations

AP-1: activator protein-1
BMPR2: bone morphogenetic protein receptor 2
CDP: CCAAT displacement protein
CREB: cyclic AMP response element-binding protein
CRS: Cis-repression site
CTCF: CCCTC-binding factor
ELK1: erythroblast transformation specific like protein
ERF: ETS2 repressor factor
ERK: extracellular signal-regulated kinase
FACT: facilitates chromatin transcription
FOXO: Forkhead box O
GFI: growth factor independence 1
HAT: histone acetyltransferase
HCK: hematopoietic cell kinase
HCMV: human cytomegalovirus
HDAC: histone deacetylase
HDACi: HDAC inhibitor
HMT: histone methyltransferase
HP1: heterochromatin protein 1
HPC: haematopoietic progenitor cell
IE: Immediate-Early
IL: interleukin
KAP1/TRIM28: KRAB-associated protein 1/Tripartite motif-containing protein 28
lncRNA
LUNA: latency unique nuclear antigen
MCMV: murine CMV
MIEP: major immediate-early promoter
MRF: myogenic regulatory factor
MSK: mitogen stimulated kinase
NF-κB: nuclear factor kappa B
NT2: NTera2
PDX1: pancreatic and duodenal homeobox 1
PKC: protein kinase C
PMA: phorbol 12-myristate 13-acetate
PML: promyelocytic leukaemia
RAR: retinoic acid receptor
SATB1: special AT-rich sequence-binding protein
Sp1/Sp3: specificity factor 1/3
SRF: serum response factor
TNFR1: TNF-α receptor 1
YY1: Yin Yang 1
